# A Therapeutic and Diagnostic Multidisciplinary Pathway for Merkel Cell Carcinoma Patients

**DOI:** 10.3389/fonc.2020.00529

**Published:** 2020-04-15

**Authors:** Marco Rastrelli, Paolo Del Fiore, Alessandra Buja, Antonella Vecchiato, Carlo Riccardo Rossi, Vanna Chiarion Sileni, Saveria Tropea, Francesco Russano, Manuel Zorzi, Romina Spina, Rocco Cappellesso, Renzo Mazzarotto, Francesco Cavallin, Franco Bassetto, Elisabetta Bezzon, Beatrice Ferrazzi, Mauro Alaibac, Simone Mocellin

**Affiliations:** ^1^Surgical Oncology Unit, Veneto Institute of Oncology IOV - IRCCS, Padua, Italy; ^2^Department of Cardiological, Thoracic, Vascular Sciences and Public Health, University of Padua, Padua, Italy; ^3^Department of Surgery, Oncology and Gastroenterology (DISCOG), University of Padua, Padua, Italy; ^4^Melanoma Oncology Unit, Veneto Institute of Oncology IOV-IRCCS, Padua, Italy; ^5^Veneto Tumour Registry, Azienda Zero, Padua, Italy; ^6^Surgical Pathology and Cytopathology Unit, Department of Medicine (DIMED), University of Padua, Padua, Italy; ^7^Department of Surgery and Oncology, Unit of Radiotherapy, Hospital Trust of Verona, Verona, Italy; ^8^Independent Statistician, Solagna, Italy; ^9^Clinic of Plastic Surgery, Department of Neuroscience, Padua University Hospital, University of Padua, Padua, Italy; ^10^Radiology Unit, Department of Imaging and Medical Physics, Istituto Oncologico Veneto IOV IRCSS, Padua, Italy; ^11^Postgraduate School of Occupational Medicine, University of Verona, Verona, Italy; ^12^Unit of Dermatology, University of Padua, Padua, Italy

**Keywords:** Merkel cell carcinoma, multidisciplinary team, therapeutic and diagnostic pathway, non-melanoma skin cancers, Merkel cancer

## Abstract

Merkel Cell Carcinoma (MCC) is a highly aggressive neuroendocrine neoplasm of the skin. Due to its rarity, the management of MCC is not standardized across centers. In this article, we present the experience of the Veneto region in the North-East of Italy, where a committee of skin cancer experts has proposed a clinical pathway for the diagnosis and treatment of MCC. Putting together the evidence available in the international literature, we outlined the best approach to the management of patients affected with this malignancy step- by- step for each possible clinical situation. Crucial in this pathway is the role of the multidisciplinary team to deal with the lack of robust information on each aspect of the management of this disease.

## Introduction

Merkel cell carcinoma (MCC) is a rare, but aggressive neuroendocrine carcinoma of the skin.

In 1972 it was described for the first time by Toker as “trabecular carcinoma of the skin” and later was renamed in its actual form due to the similar microscopic features with Merkel cells, a special type of cell found right below the epidermis (top layer of the skin). These cells are very close to the nerve endings that receive the sensation of touch and may be involved in touch. The cells also contain substances that may act as hormones. Despite this, the accurate tumor genesis is still unclear and recent works have suggested also epidermal steam cells and dermal neuroendocrine steam cells as MCC source (1–3).

According to the SEER (The Surveillance, Epidemiology, and End Results program of the National Cancer Institute) in the United States, there is a 0.7/100.000 incidence per year and this data is comparable to most of European countries (4).

Even if it is a rare tumor according to SEER program, MCC incidence has tripled during the last years with an increase of 0.8% per year and it represents worldwide the second death cause by cutaneous tumors after melanoma, with a 5-year mortality of 30% (4).

According to AIRTUM (Italian Association's Tumor Registry), there were 1400 prevalent cases of MCC in Italy in 2015. AIRTUM estimated 228 incident cases of MCC occurred in 2015 and four fifths of the patients were older than 65 years. One-year mortality was 25% and 5-year mortality was 40% (5).

MCC affects mostly elderly Caucasian men in sun-exposed areas of the body. MCC development in fact is associated mainly with UV induced mutations and Merkel cell polyomavirus (MCPyV) infection. MCC is strongly related also to immune suppression and MCC is more frequent in patients with HIV infection, transplantation, hematological malignancies, autoimmune diseases and in those treated with immunosuppressive agents (6, 7).

The complexity and rarity of the disease and the multiple comorbidities affecting most of the patients suggest the implementation of a territorial network with tertiary referral hospital with dedicated skilled specialists to whom the patients should be referred.

The aim of this study is to describe the algorithm adopted by an Italian region (Veneto) for the management of patients affected with this disease.

## Methods

The Italian National Healthcare Service (NHS) is a public system financed mainly by general taxation, and organized essentially on a regional basis (8). Its policies are grounded on fundamental values of universality, free access, freedom of choice, pluralism in provision, and equity. Health care facilities and activities are planned and organized by regional authorities in accordance with a national health plan that is designed to guarantee an equitable provision of comprehensive care throughout the country.

The Veneto Region in 2013 created an oncology network (ROV) drawn according a hub- and- spoke network design. The hub-and-spoke organization design is a model, which arranges service delivery assets into a network consisting of an anchor establishment (hub) which offers a full array of services, complemented by secondary establishments (spokes) which offer more limited service arrays, routing patients needing more intensive services to the hub for treatment (9). In many regards, the hub of the hub-and-spoke network effectively becomes a system-wide center of excellence, including centralization which concentrates resources at single sites and bolsters patient volume, fostering quality (10). The competences required for the diagnosis and treatment of MCC are comparable to the ones for the management of melanoma. Consequently the tertiary and secondary care referral hospitals are the same as indicated by Veneto region for the treatment of cutaneous melanoma in the Decree n° 118 of 08.10.2018. Oncology departments authorized by Veneto Region to prescribe Avelumab in metastatic MCC were defined with the Decree n° 129 of 31 October 2018 and are the Veneto Institute of Oncology (IOV) and Verona University Hospital (AOVR). Moreover general objectives oncological networks is to guarantee an equal and uniform accessibility to the best health care, security of services related to clinical expertise and adequate organization, timeliness in taking charge, continuity of assistance. Moreover, to increase the appropriateness prescription and provision of the network the Region defined clinical pathways (CPWs) of 39 cancer sites. CPWs are a common component in the quest to improve the quality of health, they are used to reduce variation, improve quality of care, increase equality and maximize the outcomes for specific groups of patients. Among those, a multidisciplinary team defined clinical pathways for the management of MCC (11). Finally a specific Merkel cell carcinoma needs assessment was performed calculating Veneto Region crude rates dividing the total number of cases in a given time period by the total number of persons in the population. Since the mid-nineties specific diagnostic codes have been introduced in Italy: ICD - 9 (M8246/3: neuroendocrine carcinoma and M8247/3: MCC) allowing an accurate registration of the neoplasia. The “Veneto Tumor Registry” recorded 90 incident cases of MCC from 2013 to 2015.

In Table 1 we show the crudes cases of MMC stratified by Veneto districts.

**Table 1:** Crudes cases of Merkel cell carcinoma stratified by Veneto districts.

**Residency Districts**	**Incident cases 2013–2015**	**Population**	**Annual incidence rate**
Dolomiti^a^	6	206.856	0.96
Marca Trevigiana^b^	18	885.447	0.67
Serenissima^c^	15	626.847	0.79
Polesana^d^	5	243.095	0.68
Euganea^e^	19	934.332	0.67
Pedemontana^f^	6	367.982	0.54
Berica^g^	7	499.332	0.46
Scaligera^h^	14	922.383	0.50
Veneto Region^*^	90	**4.686.274**	**0.64**

* *96% of Veneto population*.

a *Geographical area that includes the territory of Belluno*.

b *Geographical area that includes the territory of Treviso*.

c *Geographical area that includes the territory of Venezia*.

d *Geographical area that includes the territory of Rovigo*.

e *Geographical area that includes the territory of Padova*.

f *Geographical area that includes the territory of Bassano del Grappa*.

g *Geographical area that includes the territory of Vicenza*.

h *Geographical area that includes the territory of Verona*.

## Clinical Pathway

Herein we present the Veneto region proposal for the diagnosis and treatment of MCC, focusing on the central role of the multidisciplinary team in the management of this disease.

### Presentation and Initial Investigations

#### Clinical Presentation

**Figure 1:**
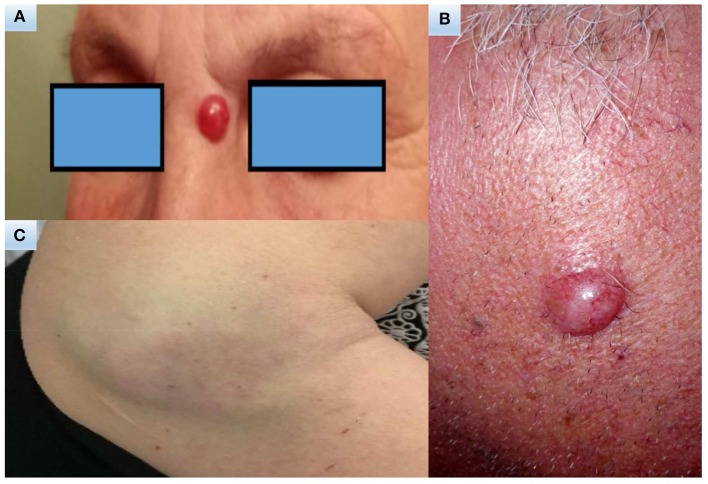
Clinical images. A 70-year-old woman with a 15-year history of polymyalgia treated with steroids **(A)**. A 65-year-old man with no significant comorbidities **(B)**. A 47-years-old woman with a 14-year history of chronic myeloproliferative syndrome initially treated with hydroxyurea for 4 years, then enrolled in a trial and treated for 7 years with ruxolitinib **(C)**.

MCC presents usually in elderly Caucasian men on sun-exposed areas of the body and head/neck is the most common site of presentation. Usually the family physician or a dermatologist are the first clinicians who come into contact with the patient and begin the diagnostic pathway. The acronym AEIOU (11) summarize the most important features: asymptomatic /lack of tenderness, expanding rapidly, immune suppression, older than 50 years, and ultraviolet-exposure. MCC in fact appears more often as a solitary, rapidly growing cutaneous red to violet nodule that might be clinically confused with other benign skin lesions, for example inflammatory lesions or cysts or with malignant tumors such as skin lymphomas, squamous cell carcinoma or metastasis (1, 4) (Figure 1).

This non-specific presentation often may lead to delayed clinical diagnosis. Unlike other cancers, the majority of tests required to confirm a MCC diagnosis occur in the primary care setting before specialist referral. The steps required for the diagnosis of MCC are the clinical evaluation and the subsequent biopsy and immunohistological analysis of the suspect lesion.

### Diagnosis, Staging and Treatment Planning

#### Histological Diagnosis

Due to the non-specific clinical presentation, the histological and immunohistochemical analysis of the suspect lesion is required to achieve MCC diagnosis. Therefore, the pathologist plays a crucial role to confirm the tumoral origin of the lesion and to rule out other tumors. Microscopically, MCC is a dermal/hypodermic-based tumor composed of nodules, sheets, and/or trabeculae of small to large, monomorphic, roundish cells with round to oval nuclei with finely dispersed chromatin (salt and pepper), indistinct nucleoli, and scant cytoplasm. Nuclear molding and crush artifacts are common. There are usually numerous mitotic figures and apoptotic bodies. Immunohistochemistry is very helpful for the diagnosis of MCC. Indeed, MCC cells are usually positive for both epithelial markers, such as AE1/AE3, CAM5.2, and CK20 (especially in the dot-like paranuclear pattern), and neuroendocrine markers, such as chromogranin, synaptophysin, CD56, and NSE. Recently, insulinoma-associated protein 1 (INSM1) has been proposed as promising marker to confirm the neuroendocrine nature of MCC (12). Most cases are also positive for neurofilament and for huntingtin-interacting protein 1 (HIP1) (13). The lack of immunoreaction for CD45 and S100 allows to exclude lymphoma and malignant melanoma, respectively, from differential diagnoses. CK7 and TTF1 are almost always negative in MCC and are useful to distinguish it from metastatic small-cell carcinoma of the lung. The pathology record should report the tumor diameter, the excision margins status, the presence/absence of lymph-vascular invasion, of extra cutaneous spreading and the coexistence of a second tumor (for example squamous carcinoma).

#### Sentinel Lymph Node Biopsy

After MCC diagnosis, the patient should be referred to the oncologic specialized referral centers, IOV (Veneto Institute of Oncology), AOPD (Padua University Hospital Center), AOVR (Verona University Hospital Center). First of all specialized clinicians should evaluate regional lymph node status to stage the disease and to define patient prognosis. In fact, lymph node status is one of the most important prognostic factors of MCC and previous studies have demonstrated that there is no primary tumor size for which the risk of nodal involvement is clinically negligible. Regional lymph nodes status should be evaluated clinically or with echography/fine needle aspiration. After that, sentinel lymph node biopsy (SLNB) can be offered to patients to assess the presence of lymph node metastasis and to stage the disease. The SLNB is performed alongside wide excision in order to avoid alterations of lymph node drainage. The pathology record should report the presence/absence of isolated tumor cells or lymph node metastasis, along with its dimension (the maximum diameter of the major tumor cell aggregate), and of the extra nodal extension of the tumor. SLNB is microscopically evaluated on multiple levels with the aid of immunohistochemistry. The SLNB-negativity is a strong predictor of longer DFS and OS in stage I and II MCC patients (14), if SLNB is positive, a complete regional lymph node dissection (CLND) must be done; positive SLN was an important prognostic factor in MCC, patients with positive SLNs had a greater risk of distant metastasis (15). If lymph nodes are clinically/echographically involved CLND can be performed instead of SLNB.

#### Imaging

Due to the rarity of the disease, there is not a defined imaging algorithm for MCC. Nowadays imaging is used mainly firstly for tumor staging, secondly for surgical and radiation planning, treatment response assessment and follow up.

Ultrasonography (US) is used to study regional lymph node metastasis. If it is positive, the patient should receive complete lymph node dissection, if negative sentinel lymph node biopsy should be performed.

If primary tumor is T4, soft tissue magnetic resonance (MRI) should be used to evaluate tumor spreading in nearby tissue.

All patients should be staged with thoracic and abdominal computerized tomography (TC), in higher risk cases with positive SLN and if there is high suspect of metastatic disease, with total body positron emission tomography (PET) with 2 deoxy 2 fluorine 18 fluoro D glucose (18 F-FDG) (16).

According to the literature, the choice of 18 F-FDG PET TC has shown benefits both in the staging phase and in the suspicious of recurrence. The 18 F-FDG PET TC led to a change in tumor staging more than a third of patients, while in the diagnosis of recurrence, highlighted unknown metastatic localizations, especially at the level of the bone with important impact on the management of MCC patients (16–18).

Brain MRI with contrast medium should be performed in all patients with T2–T4 tumors and in T1 tumors only if there is clinical suspicion of brain metastasis (19).

MCC of unknown origin should be considered stage 3 disease and staged with 18F-FDG PET TC and brain MRI (20).

#### Treatment Planning: Multidisciplinary Evaluation

All patients with a MCC diagnosis should be referred to a tertiary level hospital where they are evaluated in a dedicated multidisciplinary team with MCC skilled specialists (9).

The multidisciplinary team comprehends a care manager, a dermatologist, an oncology surgeon, a plastic surgeon, a radiation oncologist, a radiotherapist, a nuclear medicine physician, a pathologist, a medical oncologist and a psychologist.

Additional expertise or specialist services may be required for some patients with peculiar comorbidities.

In fact, patients affected by MCC frequently have multiple comorbidities usually associated with immune suppression such as past neoplasias, rheumatic diseases, autoimmune diseases, hematologic neoplasias, methabolic disease, organ transplantation and iatrogenic systemic immune suppression. All these are also risk factors for the development of MCC and require the skills and treatment of a specialized doctor (7, 8).

The multidisciplinary team nominates a care manager that is responsible of the compliance with the assisted diagnostic therapeutic pathway such us the planning of the procedures, the timing of treatment and is the point of reference for the patient (21).

Tumor stage is defined according to the eighth version of the American Joint Committee on Cancer (AJCC) staging system, effective from January 2018 and displayed in Table 2.

**Table 2:** Clinical stage group (cTNM).

**When T is**	**When N is**	**When M is**	**Then the stage group is**
Tis	N0	M0	0
T1	N0	M0	1
T2-3	N0	M0	IIA
T4	N0	M0	IIB
T0-4	N1-3	M0	III
T0-4	Any N	M1	IV

## Established Treatments

### Management of Primary Tumor and Tumor Bed

#### Surgery

Before the patient undergoes surgical treatment the multidisciplinary team should evaluate the patient general conditions and comorbidities, his past treatments (for example radiation therapy), the disease extension and the possibility of gaininig complete wound healing and functional restoration.

#### Wide Excision

Wide excision (WE) aim is to obtain negative margins to ensure that the tumor has been removed entirely. WE should be performed at the same time of SLNB. We suggest to obtain surgical margin of 1 centimeter for T1 tumors and 2 centimeters for T2-T4 tumors. If possible, the surgical wound should be closed by direct suture. If it is not possible, the plastic surgeon should be involved and he should practice a flap surgery or skin graft only if there are free margins and SLNB has been performed. Choosing the type of surgical reconstruction should not retard the adiuvant radiation therapy (22).

#### Radiation Therapy

Radiation therapy can be used as radical, adjuvant, palliative treatment due to MCC sensibility to radiotherapy. Several studies have suggested that all patients should receive post-operative adjuvant radiation therapy to the primary site, which is associated with a reduced risk of local recurrence. Doses can vary according to margins status after wide excision: 50–60 Gray (Gy) with free margins, 56–60 Gy with positive margins, 60–66 Gy with macroscopically positive margins. The median fraction size is 2 Gy. Patient should receive adiuvant radiation therapy as soon as possible after surgical treatment (23, 24). A recent systematic review excludes a benefit of adjuvant radiotherapy in pathologically node-negative patients with very small primary tumors (<2 cm, stage I) with free margins and no risk factors. In the absence of a clear and well-defined standard algorithm of adjuvant RT in MCC treatment in presence of unfavorable factors such as lymphatic- vascular invasion, immunosuppression condition, lymphoproliferative diseases, location of the primary tumor in the head and neck area, absence of a correct assessment of the pathologically lymph node status, the approach must be personalized after discussion by the multidisciplinary team (25).

Patients considered medically or technically inoperable and of good performance status should receive radiation therapy alone with a dose of 60–66 Gy in 30–33 daily fractions with wide fields encompassing macroscopic disease with a high likelihood of achieving locoregional control (24).

Regional lymph nodes basin radiotherapy should be offered if lymph node basin is clinically positive and if SLNB has not been performed. There is a limited number of studies that compare the efficacy of CLND and lymph node radiation therapy because radiotherapy is usually offered as best treatment to avoid local recurrence after CLND (25).

### Management of Regional Lymph Nodes

Lymph nodes should be pathologically evaluated in almost every case of MCC (14, 15).

If the lymph nodes are clinically positive, the treatment options comprehend CLND and/or regional radiotherapy.

If the lymph nodes are clinically negative, SLNB should be considered and planned at the same time as the wide local excision because clinical occult nodal micrometastases are present in one third of patients, regardless of size of the primary tumor. If occult tumor metastasis is detected the patients should receive CLND and/or elective regional radiotherapy to the draining lymph node basin, but none of these have been compared in a randomized fashion (14, 15, 26).

In about one-third of patients with MCC of the neck the regional lymph nodes are involved (stage III disease). This includes cases detected at SLNB, cases with clinically-detected and confirmed involvement of the regional lymph nodes or in-transit metastases (27).

In the patients with positive regional nodes, 5-year survival was 39.7%, in those with clinically detected disease was 26.8%, and in those with in-transit disease was 41.1%. In the patients with an unknown primary (3.6%) 5-year overall survival was 42.2% (15).

Treatments include lymphadenectomy with or without radiotherapy, or definitive radiotherapy to the regional lymphnodes. In patients who have neck dissections (stage IIIB disease) if there is extracapsular spread or multiple nodes are involved, adjuvant radiotherapy is highly recommended.

When the SLNB is positive but there is not evidence of distant metastatic disease, the definitive treatment of the regional lymphnodes is indicated and may be followed by adjuvant radiotherapy.

CLND should extend to nodal levels in which there is a high risk of occult nodal disease, include involved parotid tissue and involved levels in the neck (27).

### Management of Metastatic Disease

#### In Transit Metastasis

In transit metastasis treatment options include surgery, isolated limb perfusion (ILP), electrochemotherapy and radiation therapy.

Radical surgery is indicated when there is a limited number of cutaneous metastasis treatable with conservative operation.

Radiation therapy represents the first choice treatment for in transit metastases that cover a wide area of head/neck and are not surgically operable. The suggested dose is 60–66 Gy (28).

ILP represents the gold standard treatment for extremities in transit non-operable metastasis (29).

Electrochemotherapy is the best choice for head/neck and trunk in transit metastasis not suitable for surgical or radiotherapy treatment. Extremities in transit metastasis can be treated with electrochemotherapy when ILP cannot be performed or after ILP (30).

#### Metastatic Disease: Surgery and Systemic Treatment

In advanced metastatic disease surgery may represent an effective treatment option, in particular for patients with skin metastases and for metachronous oligometastatic situations with long free intervals.

Before undergoing surgery, the specialized surgeon should consider the number of metastasis, the site of metastasis, the number of involved organs, the disease- free survival and the patient performace status.

#### Chemotherapy

In advanced MCC cytotoxic chemotherapeutics are commonly used even if responses are rarely durable and high toxicity is shown. The most common regimes use a platinum-based product plus etoposide. Second line therapy comprehends cyclophosphamide, doxorubicin, and vincristine.

A recent systematic literature review, which included 33 chemotherapy studies for metastatic MCC, reported that platinum was used in two thirds of cases, with or without etoposide, and platinum-free regimens in the remaining third. Excluding case reports and case series and considering five retrospective studies/reviews, the percentage of objective tumor responses was 52–61% on the first line and 23% on the second chemotherapy line. Regardless of the line, the response duration was <6 months and the progression free survival (PFS) was 3 months in the first line and 2 months in the second line. Finally, overall survival (OS), reported in two of the five studies, was around 9 months (31, 32).

#### Palliation Therapy

Nowadays a large part of advanced MCC carcinoma treatment remains palliative. The multidisciplinary team should cooperate in order to improve the patient quality of life. In fact, in poor prognosis cancers early referral to palliative care can improve the quality of life and in some cases may be associated with survival benefits (33).

### Emerging Strategies

#### Immunotherapy and Target Therapy

The high frequency of association of MCC with MCPyV, the correlation of the prognosis of MCC with immunosuppressive states, the high mutational load in the negative MCPyV MCC and the expression of markers such as PD-L1 in tumor tissue and in the leukocyte microenvironment peritumor of patients with MCC led to experiment with inhibitors of new immune checkpoints in MCC. Pembrolizumab, Avelumab, and Nivolumab have been studied both in patients pre-treated with chemotherapy and in patients who had not received previous chemotherapy. The percentage of objective tumor responses was 56–73% in the first line and 33–50% in the second, PFS was 17 months in the first line and 3 months in the second, OS not achieved in the first and 13 months in the second (34–36). PFS in the first-line pembrolizumab study was 17 months, with two thirds of patients alive at 2 years, far superior to historical chemotherapy results (34). In the study published with Avelumab in patients pre-treated with chemotherapy, although the percentage of objective responses and the PFS were almost comparable to those known for chemotherapy, in more than two thirds of the responsive patients the response lasted at least a year, done completely new and much more favorable than what is known for chemotherapy, which must be added to a 13-month OS with Avelumab compared to almost six for chemotherapy. Note that 36% of patients are alive at 2 years (37). Based on this study, in 2017 the FDA approved Avelumab for metastatic MCC regardless of the line of therapy (38). Results of a planned analysis of part B of the Javelin study (36), which refers to a group of 39 patients with metastatic MCC treated with Avelumab in the first line.

Clinical activity was assessed in 29 patients who had a minimum follow-up of 3 months. A confirmed objective response rate of 62% was observed; responses were ongoing at the time of analysis in 78% of cases, with a response duration of 6 months equal to 83%. Both in the first and second line the results of the studies showed a plateau of PFS, which is reached around 10–11 months, demonstrating that responding malignancies tend to maintain the response. Therefore, with immunotherapy, unlike with chemotherapy, the problem remains primary resistance, while secondary resistance seems negligible. In other words, the tumor progression after the first year is relatively low. When performing a historical comparison with chemotherapy for Avelumab, a percentage of PFS of 29% at 1 year and 26% at 2 years is noted, compared to 5 and 0%, respectively, for chemotherapy. The data of clinical activity and efficacy of Avelumab in the first line and in patients pretreated with chemotherapy, compared with the historical cases of patients with metastatic MCC treated with chemotherapy, suggest that Avelumab is more effective than chemotherapy in terms of PFS and OS, and which could have greater efficacy when used in the first line. The toxicity profile of the three immunotherapy drugs proved to be superimposable and in line with what is known from experience in other sectors of oncology. The response to anti-PD-1/PD-L1 was not significantly different in relation to some potential predictive factors, such as PD-L1 + vs. PD-L1–, MCPyV + vs. MCPyV–, high mutation load vs. low (38).

### Psycho-Oncological Aspects

The Patients with MCC, are very fragile patients, have factors strongly associated with the development of the disease, which include older age (more than 65 years), immune suppression, presence of comorbidity, chronic use of immunosuppressive (kidney/heart/liver-transplant patients) and/or immunomodulating drugs.

According to a recent study,the patients with MCC have no symptoms and pain associated with the disease before and after diagnosis, overall they carry on a normal daily life, and still have all their physical abilities. In contrast uncertainty about the future creates a substantial psychological impact on patients and families (39).

The detection of health-related quality of life (HRQoL) in patients with MCC does not yet have validated disease-specific tools, therefore the questionnaires EQ-5D and EORTC QLQ-C30 are widely used.

The need to evaluate HRQoL in patients with Merkel enrolled in an important clinical trial, has led to make up for this lack with the Functional Assessment of Cancer Therapy - Melanoma (FACT-M) (40, 41).

The FACT-M is validated in patients with melanoma, but the same shared characteristics can perform the correct use of this scale for MCC patients.

### Follow Up

Half Patients affected by MCC develop lymph node metastasis and one-third distant metastasis (42).

During the patient evaluation immune suppression conditions should be taken under consideration, in fact immunosuppressed MCC patients are at higher risk of recurrence and MCC-related death (6, 7).

Follow up visits comprehend clinical and imaging examination and are performed every 3–6 months for the first 3 years and every 6–12 months till the fifth year. After that, for other 5 years, the patient is visited once a year and imaging examination is performed only on the basis of clinical suspicion.

The first 3 years of follow up are crucial because 90% of patients develop recurrence during the first 2 years.

In stage I and IIa disease follow up visits are performed every 6 months for the first 3 years with clinical and imaging examination that can comprehend 18F-FDG PET TC, abdominal and thoracic TC, regional lymph node echography, abdominal echography, thoracic X-RAY.

In stage IIb, IIIa, IIIb disease follow up visits are performed every 3 months for the first 3 years and imaging examination comprehends also brain MRI (19).

## Discussion

Though MCC remains a rare tumor, its incidence has risen rapidly over the last decades and represents the second most common cause of skin-cancer death after melanoma. Nowadays surgery and radiation therapy are the standardized treatment for local and regional control of the disease, whereas chemotherapy is reserved for metastatic disease. Several trials of immune and targeted molecular therapies are ongoing and may provide further treatment options for patients with advanced MCC in the near future. Because of the rarity of the disease, multi institutional collaborative efforts represent the future of MCC management in order to create clinical trials and universal diagnostic and therapeutic guidelines. Moreover, due to the complexity of patients affected by MCC, often affected by immune comorbidities or immune suppression, a multidisciplinary approach is the key in MCC management in order to provide patients with a tailorade treatment wich can maximize the chances of survival.

## Data Availability Statement

The datasets generated for this study are available on request to the corresponding author.

## Ethics Statement

This study was carried out in accordance with the recommendations of Ethics Committee for the Clinical Trial of Veneto Institute of Oncology (CESC-IOV) with written informed consent from all subjects. All subjects gave written informed consent in accordance with the Declaration of Helsinki. The protocol was approved by the above mentioned Ethics Committee for the Clinical Trial of Veneto Institute of Oncology (CESC IOV).

## Author Contributions

MR, SM, BF, and PD contributed conception and design of the study. BF, SM, and AB wrote the first draft of the manuscript. PD and BF wrote sections of the manuscript. All authors contributed to manuscript revision, read, and approved the submitted version.

### Conflict of Interest

The authors declare that the research was conducted in the absence of any commercial or financial relationships that could be construed as a potential conflict of interest. The reviewer PQ declared a past co-authorship with several of the authors CR, MA, and SM to the handling editor.
